# Commercialism

**Published:** 1921-12

**Authors:** 


### COMMERCIALISM

Among the echoes following the meeting of the National Dental Association, resulting not from official debate but from informal or incidental discussion of things in general, comes one relating to *commercialism.*

There is no mistaking the underlying current of thought and feeling that has existed for some time past and which is growing steadily, against the apparent tendency toward commercializing the development of ideas in dental practice, which has in the last few years been making considerable headway in the West especially, where most anyone with a scheme, original or otherwise, or a bit of special technic for sale, seems to think a ready and easy market awaits him.

It is not the intention to criticize a man for desiring to create a revenue from his perfected knowledge or skill through teaching others, but there is another and broader viewpoint of the matter which those who are anxious to impart instruction at a stipulated monetary consideration, only, seem to be blind too. There is no justifiable reason for fostering and encouraging the purely mercenary among our prominent clinicians and lectures. If their ideas are not worthy enough and their feelings toward their chosen profession not broad enough to bring the fruits of private research and study before a regularly organized convention of professional brethren, with honor and distinction as the rewards of legitimate effort; if personal interest in high standards and scholarly advancement for the profession as a whole is not to be seen, except for a price, the chances are a hundred to one that they have nothing worth while to exhibit.

The "Post Graduate Meeting" as we know it in the West has been an excellent thing in many ways but the tendency to convert our regular society meetings into post graduate classes, at so much per head, when every member of the organization is or should be eligible to "sit in" at every session during the entire time of the meeting and get value received for his regular dues is, as we look at it, a serious mistake and in the long run will do the organization no good.

When a member of a dental society shows enough interest and enthusiasm to dismiss business, close his office for a week to attend the meeting of his society, often held at a considerable distance from home, that meeting in every department should be wide open to him, there should be no further fee extracted or expected from him. No private counter attraction should be allowed to absorb a single minute of the regular convention time.

If we are to have this post graduate work with the additional fee and there is much of it that is worthy, why not make it a separate affair and run it entirely outside and distinct from the regular meetings of the dental society, where every clinic and lecture or exhibit should be held for the sole benefit of each and every member of the society in good standing, his dues constituting the only fee.

If, on the other hand, our society dues seem insufficient to support first-class programs at our meetings and make them both interesting and instructive, why not raise them to that point?—*Northwestern Journal.*

